# Hydrophilic loop 1 of Presenilin-1 and the APP GxxxG transmembrane motif regulate γ-secretase function in generating Alzheimer-causing Aβ peptides

**DOI:** 10.1016/j.jbc.2021.100393

**Published:** 2021-02-08

**Authors:** Lei Liu, Bianca M. Lauro, Michael S. Wolfe, Dennis J. Selkoe

**Affiliations:** 1 Ann Romney Center for Neurologic Diseases, Department of Neurology, Brigham and Women’s Hospital, Harvard Medical School, Boston, Massachusetts, USA; 2 Department of Medical Chemistry, University of Kansas School of Pharmacy, Lawrence, Kansas, USA

**Keywords:** γ-secretase, Aβ, presenilin-1, γ-secretase modulator, Alzheimer’s disease, Aβ, amyloid-beta, AD, Alzheimer’s disease, APP, amyloid precursor protein, CM, conditioned media, DMEM, Dulbecco’s modified Eagle’s medium, FAD, familial Alzheimer’s disease, FBS, fetal bovine serum, GSM, γ-secretase modulator, HL-1, hydrophilic loop-1, HMW, high-molecular-weight, PS1, presenilin-1, TMD, transmembrane domain

## Abstract

γ-Secretase is responsible for the proteolysis of amyloid precursor protein (APP) into amyloid-beta (Aβ) peptides, which are centrally implicated in the pathogenesis of Alzheimer’s disease (AD). The biochemical mechanism of how processing by γ-secretase is regulated, especially as regards the interaction between enzyme and substrate, remains largely unknown. Here, mutagenesis reveals that the hydrophilic loop-1 (HL-1) of presenilin-1 (PS1) is critical for both γ-secretase step-wise cleavages (processivity) and its allosteric modulation by heterocyclic γ-modulatory compounds. Systematic mutagenesis of HL-1, including all of its familial AD mutations and additional engineered variants, and quantification of the resultant Aβ products show that HL-1 is necessary for proper sequential γ-secretase processivity. We identify Y106, L113, and Y115 in HL-1 as key targets for heterocyclic γ-secretase modulators (GSMs) to stimulate processing of pathogenic Aβ peptides. Further, we confirm that the GxxxG domain in the APP transmembrane region functions as a critical substrate motif for γ-secretase processivity: a G29A substitution in APP-C99 mimics the beneficial effects of GSMs. Together, these findings provide a molecular basis for the structural regulation of γ-processivity by enzyme and substrate, facilitating the rational design of new GSMs that lower AD-initiating amyloidogenic Aβ peptides.

The generation of amyloid β-peptides (Aβ) *via* sequential cleavages of APP by β-secretase (BACE1) and the presenilin/γ-secretase complex is central to the initiation of Alzheimer’s disease (AD) (1, 2, 3). γ-Secretase is a high-molecular-weight (HMW) multiprotein complex with 20 transmembrane domains (TMDs) and has an unusual intramembrane di-aspartyl catalytic site within the presenilin component. Extensive biochemical, cell biological, and structural studies of presenilin suggest that γ-secretase processing requires at least six steps to achieve hydrolysis of the peptide bonds of a transmembrane substrate: 1) initial γ-secretase activation through the auto-proteolysis of presenilin-1 or -2 (PS1, PS2); 2) substrate TMD docking to γ-secretase; 3) substrate TMD unwinding and binding to the di-aspartyl active site; 4) the initial endopeptidase (ε) cleavage of the substrate, leading to either Aβ48 or Aβ49 production line; 5) successive carboxypeptidase-like (γ) cleavages (*i.e.*, “trimming” or “γ-processivity” (4)); and 6) release of the final peptide products. The hydrophilic loop-1 (HL1) between TMD 1 and 2 of PS1 or PS2 is a key region that appears to participate in substrate docking (5), γ-processivity, and the binding of γ-secretase modulators (GSMs) (6) that enhance processivity. Moreover, HL-1 is a hot spot of familial Alzheimer’s disease (FAD) PS1 mutations (20 known AD-causing mutations within 34 residues) (https://www.alzforum.org/mutations).

Although recent advances in cryo-EM analyses of γ-secretase have provided important structural information about the relative positions of the PS1 TMDs and many of their individual amino acids (7, 8, 9), the limits of resolution of cryo-EM to date preclude conclusions about the fine biochemical details of PS1 residues and how they contribute to PS1 enzymatic function. Moreover, recent cryo-EM modeling of a substrate (APP) bound to the protease (PS1) was based on cystine cross-linking between artificial mutations PS1 Q112C within HL-1 and APP-C83 V8C (10), suggesting that HL-1 would likely have been constrained in a nonnative conformation in this engineered, covalently bound complex. In addition, one of the two catalytic aspartates of PS1 had necessarily been mutated to an inactive alanine to stabilize the artificial complex. Therefore, a detailed biochemical study of this HL-1 region is essential to further our understanding of native γ-secretase enzymatic function. To this end, we have analyzed all individual residues (aa 101–120) of HL-1 of PS1 with regard to 1) the enzymatic activity of the FAD mutations therein as well as the effects of alanine scanning mutagenesis (Ala substitutions at each residue within this region); 2) the performance of five γ-secretase modulators (GSMs) of two general classes on Aβ generation from the HL-1 FAD-mutant residues; and 3) the response of these GSMs to the Ala substitutions. Moreover, we explored the mechanism of GSMs by examining the function of the GxxxG domain in the APP transmembrane region and found that G29 (Aβ numbering) is a key residue for proper γ-secretase processivity. The combined data provide unexpected mechanistic details of the structural regulation of γ-processivity in a way that should facilitate the rational development of novel GSMs for treating and preventing AD.

## Results

### Aβ generation profiles of FAD mutations in the PS1 HL-1 domain correlate strongly with clinical ages of onset

**Figure 1:**
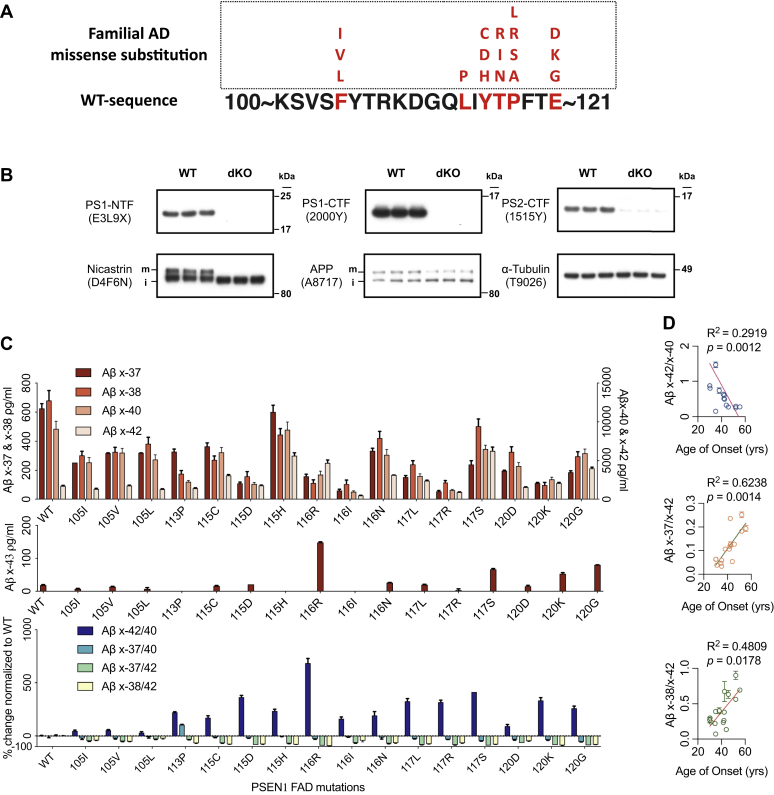
**Profile of secreted Aβs from cells expressing PS1 FAD mutants.***A*, sequence of PS1 HL1 region; *red*: sites at which known FAD mutations occur; (*B*) Wt HEK293 and PS1/2 dKO cell lysates blotted for the indicated proteins; (*C*) Aβs measured by ELISA from CM of dKO cells coexpressing wt or mutant PS1 with wt APP (means ± SD, n = 2). *Upper panel*: *left* Y-axis: Aβx-37 and -38, *right* Y-axis: Aβx-40 and -42; (*D*) Correlations between specified Aβ ratios and the FAD patients’ age of clinical onset (AOO) for 16 HL-1 mutations; nonparametric Spearman correlation.

Taking advantage of our new, sensitive immunoassays (11) that detect and quantify virtually all secreted Aβ C-terminal variants (Aβ43, 42, 40, 38, and 37), we were able to quantify γ-secretase processivity to identify the pathogenic profiles of all FAD PS1 mutations located in HL-1 (aa 101–120; see protein sequence in Fig. 1*A*). First, we used CRISPR/Cas9 on HEK293 cells to generate PS1/2 double knockout (dKO) cells in order to eliminate endogenous PS activity. The best dKO line obtained had no detectable PS1-NTF or PS1-CTF and almost no PS2 expression (Fig. 1*B*); as a result, the proper maturation (N+O-linked glycosylation) of the nicastrin (NCT) component of γ-secretase was prevented, as expected (12) (Fig. 1*B*). Next, we generated PS1 constructs for all 17 known FAD mutations within the HL-1 region (see amino acid sequence in Fig. 1*A*) and transiently coexpressed them in the HEK293 PS dKO line together with wt human APP. We then analyzed the resultant conditioned media (CM). Quantification of the secreted Aβ species in CM showed that compared with wt-PS1, 1) 15 of 16 FAD mutations decreased total secreted Aβs (*i.e.*, combining the 43, 42, 40, 38, and 37 peptide values); 2) all 16 mutations increased the Aβ42/40 ratio in the CM, as expected; and 3) all 16 mutations decreased Aβ37/40, Aβ37/42, and Aβ38/42 ratios (Fig. 1*C*). Among these, the most severe mutation, T116 R, a) increased Aβ42/40–684% of that in wt PS1 transfectants; b) decreased Aβ37/42 and Aβ38/42 by 90% and 94% below wt, respectively; and c) increased Aβ43 eightfold of the wt level. Moreover, the proteolytic shifts underlying the changes in ratios differed mechanistically among the various FAD mutations compared with wt PS1. For example, 1) Y115H increased Aβ42 production at the expense of Aβ37 and 38 while Aβ40 production remained the same as wt-PS1; 2) Y115H resulted in 21.9% more total secreted Aβ peptides than with wt PS1; 3) T116R increased Aβ42 and 43 production at the expense of Aβ37, 38 and 40; 4) T116R led to 31.5% less total Aβ secreted than did wt PS1; and 5) P117S increased Aβ42 and 43 production at the expense of Aβ37 and 40, but Aβ38 was only moderately decreased by 26%. Considering the complexities in PS1:APP conformational changes that could alter the sequential cleavages, there could be 1) a “product line” switch between Aβ48 and Aβ49 as the initial ε-cleavages of the APP TMD; 2) reduced processivity toward the final products Aβ37 and Aβ38 through a loosened interaction between PS1 enzyme and APP substrate. Moreover, in a prior report (11), we tested a HEK293 line stably expressing Y115H and observed similar elevated Aβ42/40 ratios: 0.71 for that stable line and 0.63 for the current transient transfectant, so this markedly increased Aβ42/40 ratio from Y115H occurred consistently across experiments. To our knowledge, such altered profiles of γ-processivity among FAD-causing PS1 mutants (Fig. 1*C*) are detailed here for the first time, namely the measurement of all five C-terminal variants of secreted Aβ to unveil the diverse effects of PS1 mutations.

Of special pathogenic significance, the Aβ42/40, Aβ37/42, and Aβ38/42 ratios each correlated strongly and significantly with the reported age of onset (AOO) of clinical impairment across these 16 FAD mutations (Fig. 1*D*). The tightest correlation occurred with the Aβ37/42 ratio, which comprises the ratio of the shortest to the second longest secreted Aβ peptides, thus ideally reflecting the efficiency of progressive carboxypeptidase trimming (processivity) by PS1 (Fig. 1*D*). These findings contrast with an earlier study of 138 FAD mutations that used an *in vitro* biochemical reaction; the authors did not find a correlation of Aβ42/40 to AOO when recombinant γ-secretase complexes (with wt versus mutant PS1) were used to generate Aβ peptides from recombinant APP substrates *in vitro* (13). We speculate that the discrepancy arises because the *in vitro* paradigm is not an optimal system to analyze a range of FAD mutations, in that 68 out of 138 mutant recombinant PS1 enzymes the authors tested showed <10% of the Aβ40 + 42 production than did wt PS1/γ-secretase (13). This cleavage inefficiency suggests that a reconstituted *in vitro* γ-secretase assay system is not sufficiently native and robust to discern pathogenically meaningful differences among many PS FAD mutations.

In contrast, in our *in vivo* cellular assay system, we were able to compare almost all secreted Aβ peptides, allowing us to observe a strong correlation between the patients’ AOO and three different Aβ ratios that reflect the details of γ-processivity. This system will be expanded to test more mutations in other regions of PS1 to further validate our findings. Here, we continued to explore the HL-1 region of PS1.

### HL-1 region of PS1 is critical for proper γ-processivity

**Figure 2:**
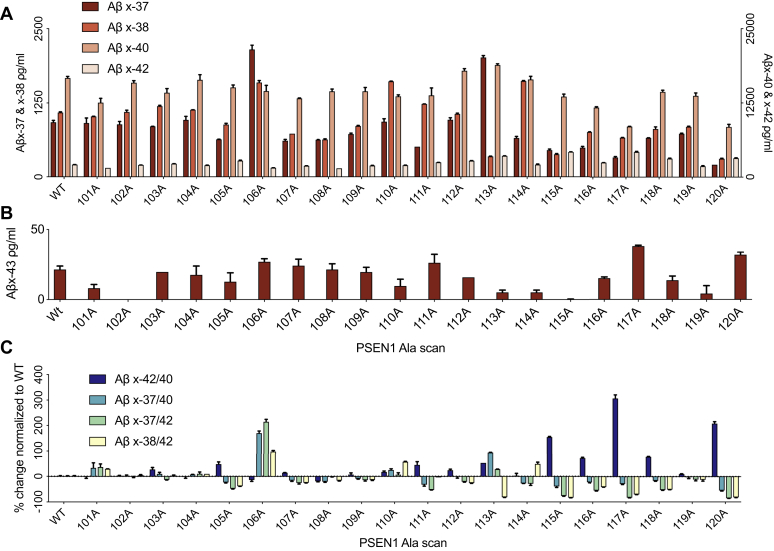
**Profile of secreted Aβs from cells expressing Ala-substituted HL-1 PS1 variants.***A* and *B*, Aβs measured by ELISA from CM of dKO cells coexpressing wt or Ala-mutant PS1 plus wt APP (means ± SD, n = 2). *A*, *left* Y-axis: Aβx-37 and 38, *right* Y-axis: Aβx-40 and 42. *C*, Aβ peptide ratios.

Next, to deeply probe the structure:function relationship of the HL-1 region, we generated a library of variants that change each amino acid of HL-1 from residue 101 to 120 into alanine(Ala) and expressed these Ala mutants in the PS1/2 dKO HEK cells along with wt-APP. Analysis of secreted Aβs in the CM of these transient transfectants in the dKO cells (Fig. 2, *A–C*) showed that: 1) eight variants have Aβ production profiles similar to that of wt-PS1 (K101A, S102A, V103A, S104A, R108A, K109A, D110A, E119A), which is reasonable as none of these eight variants are known to be pathogenic; 2) 12 variants have different Aβ production profiles than wt-PS1 and can be categorized into three groups having increased processivity (Y106A), decreased processivity (F105A, T107A, G111A, Q112A, Y115A, T116A, P117A, F118A, E120A,), or other (L113A, I114A). Then, we analyzed each group to interrogate the mechanism for these diverse enzymological effects.

Compared with wt PS1, Y106A underwent enhanced γ-processivity as reflected by: a) no change in total secreted Aβ species: the sum of Aβ42 + 40 + 38 + 37 from Y106A decreased only 4.7% versus wt levels; b) decreases in Aβ42 by 25.8% and Aβ40 by 13.1%; and c) increases in Aβ37 by 132.3% and Aβ38 by 46.6%. Thus, for Y106A, the Aβ42/40 ratio decreased 14% while the Aβ37/40, Aβ37/42, and Aβ38/42 ratios increased to 169%, 214%, and 98% of wt PS1 transfectants, respectively. Next, we found that Ala substitutions F105A, T107A, G111A, Q112A, Y115A, T116A, P117A, F118A, and E120A all lead to different degrees of reduced γ-processivity, as shown by increased Aβ42/40 ratio and decreased Aβ37/40, Aβ37/42, and Aβ38/42 ratios simultaneously. Interestingly, P117A is actually an FAD mutation, and accordingly, it showed the strongest reduction in γ-processivity among all of our 20 Ala substitutions (Fig. 2*C*). Moreover, four of these nine variants having decreased γ-processivity were at residues harboring FAD mutations. Within the third category, L113A showed partially enhanced γ-processivity along the Aβ 49–46–43–40–37 product line, as reflected by a) increasing the total level of secreted Aβs by 19.1%; b) increasing Aβ37 to 218% and Aβ40 to 112% of wt PS1 production levels, while decreasing Aβ43 to 25% of the wt PS1 level; and c) inhibiting the Aβ 48–45–42–38 product line to yield decreased Aβ38 to 31.6% and increased Aβ42 to 171.3% of wt PS1 (Fig. 1*C*). The distinct effects of substituting Y106 vs. L113 with alanine exemplify the complex regulation of γ-processivity by the fine structure of presenilin, revealing distinctive pathways of tri-, tetra-, and penta-peptide trimming. Meanwhile, I114A showed a change of end-products between Aβ37 and Aβ38, in which I114A increased Aβ38 production and decreased Aβ37, without changing Aβ40 or 42 (Fig. 2*A*). To expand our discovery regarding the criticality of Y106 and L113 for γ-processivity, we generated six more substitutions for Y106 (P, T, R, E, F, or L) and for L113 (Q, T, R, E, F, or I). Using the same experimental setup, we show in Fig. S1*A* that among these 12 new variants, only Y106T could obviously enhance γ-processivity, with an increment of Aβ38/42 ratio by 136.7% compared with wild-type PS1. From the seven total substitutions at Y106 and L113 respectively, it is difficult to observe a pattern to provide more information on the structure–activity relationship, besides the general importance of the two natural residues (Y; L) for γ-processivity. In addition, we found that Ala variants at the C-terminal region of HL1 (116, 117, 118, 119, and 120) significantly reduced γ-processivity, which concurs with these residues being hot spots for FAD mutations (Fig. 1*A*). The latter findings suggest that γ-processivity is also sensitive to changes in the conformation of the C-terminal region of HL1.

Collectively, the detailed findings from FAD mutants and alanine scanning mutagenesis indicate that the HL-1 region has a major modulatory effect on proper γ-processivity, in accord with certain prior reports (5, 6, 14).

### HL-1 region of PS1 is critical for γ-secretase modulation

**Figure 3:**
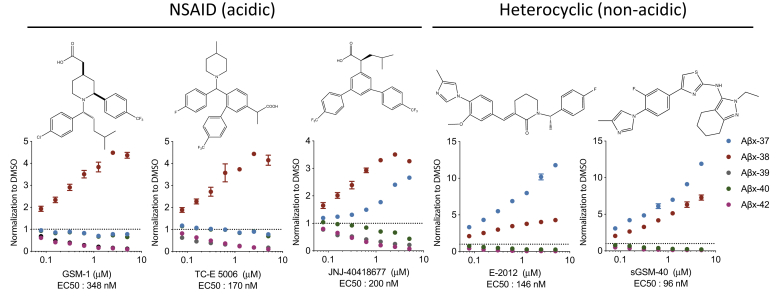
**Performance of two types of GSMs.** Aβs (42, 40, 39, 38, and 37) measured by ELISA from CM of sw-APP cells treated with serial dosages of GSMs. Data was normalized to DMSO vehicle control. n = 3.

We next examined whether HL-1 is the responsible part of PS1 for the effects of γ-secretase modulators (GSM), as prior evidence has shown that Y106 could serve as a binding site of PS1 to E2012, a potent heterocyclic GSM developed by Eisai. Since the surprising discovery of sulindac sulphide and two other nonsteroidal anti-inflammatory drugs (NSAIDs) that reduced Aβ42 production from cells (15), hundreds of compounds have been generated to modulate γ-secretase activity allosterically with the goal to enhance γ-processivity toward the shorter end products, Aβ 38 and 37, an effect similar to that of Y106A, which we observed above. Current GSMs are divided into two broad classes of compounds: the NSAID (acidic) type and the heterocyclic (nonacidic) type (16). We tested the performance of three acidic GSMs (GSM-1, TC-E 5006, and JNJ-40418677) and two heterocyclic GSMs (E2012 and sGSM-40) (17) (Fig. 3). Analysis of the abundantly secreted Aβs generated by sw-APP cells (HEK293 cells stably expressing the “Swedish” FAD-causing APP mutation) was accomplished by treating these cells with serial doses of each of these five GSM compounds. All five GSMs decreased Aβ42 and 39 while increasing Aβ38 levels, compared with DMSO-treated sw-APP cells. The two nonacidic (heterocyclic) GSMs and JNJ-40418677 also decreased Aβ40 and increased Aβ37, whereas GSM-1 or TC-E 5006 did not (Fig. 3), consistent with a previous report (18). These distinct effects on Aβ profiles suggest differential structural modulation of PS1 by different GSMs.

**Figure 4:**
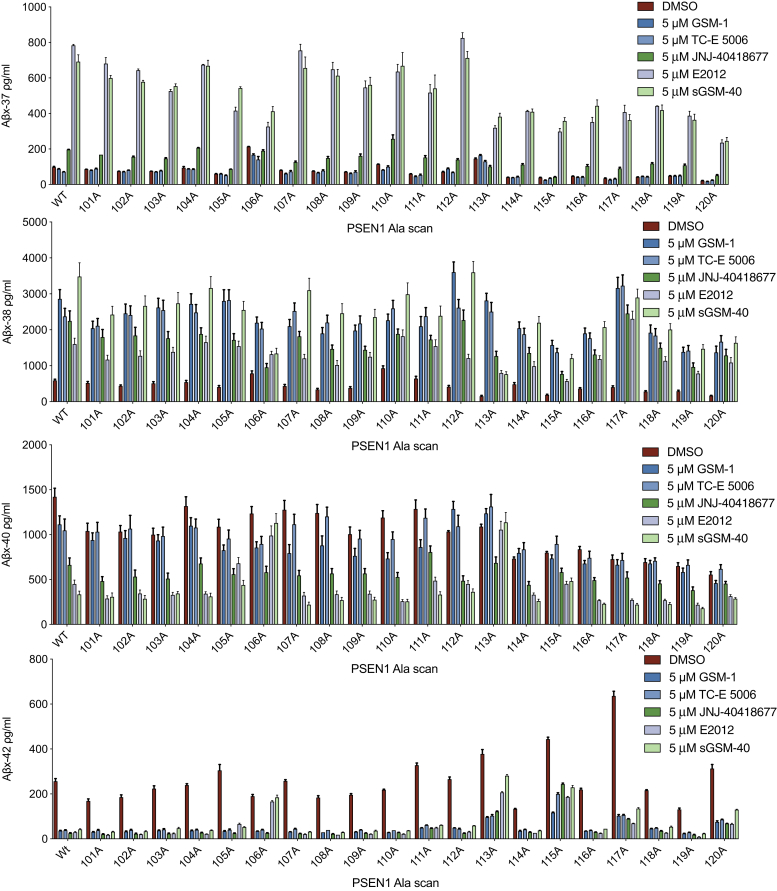
**Profile of Aβs secreted from cells with Ala-substituted PS1 variants treated with GSMs.** Aβ ELISAs on CM of dKO cells coexpressing PS1 wt or Ala-variants plus wtAPP after 12 h treatment with the GSMs (means ± SD, n = 2).

To discern the maximal effects of the GSMs, we applied each at 5 μM [the highest dose tested in the sw-APP cells (Fig. 3)] to dKO 293 cells coexpressing wt-APP with each of our Ala-substituted HL-1 variants and collected the CM for 12 h. Analysis of the four secreted Aβ peptides (Aβ37, 38, 40, 42) showed that Y106A blunted the modulatory effects of the nonacidic but not the acidic GSMs as regards decreasing Aβ42 and 40 and increasing Aβ37 and 38 (Fig. 4). A prior report had studied E2012 effects on a deletion of residues 101–110 (11); our results more precisely demonstrate that Y106 is a critical functional residue for not only E2012 but also another nonacidic (heterocyclic) modulator, sGSM-40, whereas two of the acidic GSMs, GSM-1 and TC-E 5006, but not JNJ-40418677, still worked on Y106A. Substitutions L113A and Y115A also subtly but significantly blunted the modulatory effect of the nonacidic GSMs, as reflected by the changes in the Aβ42/40 and Aβ37/42 ratios upon treatment (Fig. S1*B*). Thus, PS1 variants responded to five GSMs differently, suggesting that GSMs have multiple binding sites or orientations. Y106, L113, and Y115 could be residues critical for heterocyclic-type GSM binding to PS1. Previous studies suggested that GSM-1 and another acidic GSM, NS-1017, could bind to the TMD-1 of PS1 to confer modulatory activity (19). To test this hypothesis, we also generated a library of variants that substitute alanines (Ala) into residues 85–99 of the transmembrane domain-1 (TMD-1) region. We expressed these ten Ala-substituted variants with wt-APP in PS1-dKO cells and collected the CM for 12 h of treatment with 5 μM of each GSM. Analysis of Aβ42 secreted from these cells showed that none of the ten Ala variants blunted the effects of the five GSMs (Fig. S2). From these data, TMD1 is unlikely to be the binding site of these 5 GSMs, including GSM-1, which is reasonable in that a highly helical and hydrophobic transmembrane region is less exposed to extracellular compounds compared with the HL-1 region. Although we have little knowledge of the binding sites for acidic GSMs, our findings above do indicate the critical roles of the HL-1 region of PS1 for γ-processivity and response to heterocyclic GSMs.

### GxxxG domain of the APP transmembrane region, especially G29, is critical for γ-secretase processivity

**Figure 5:**
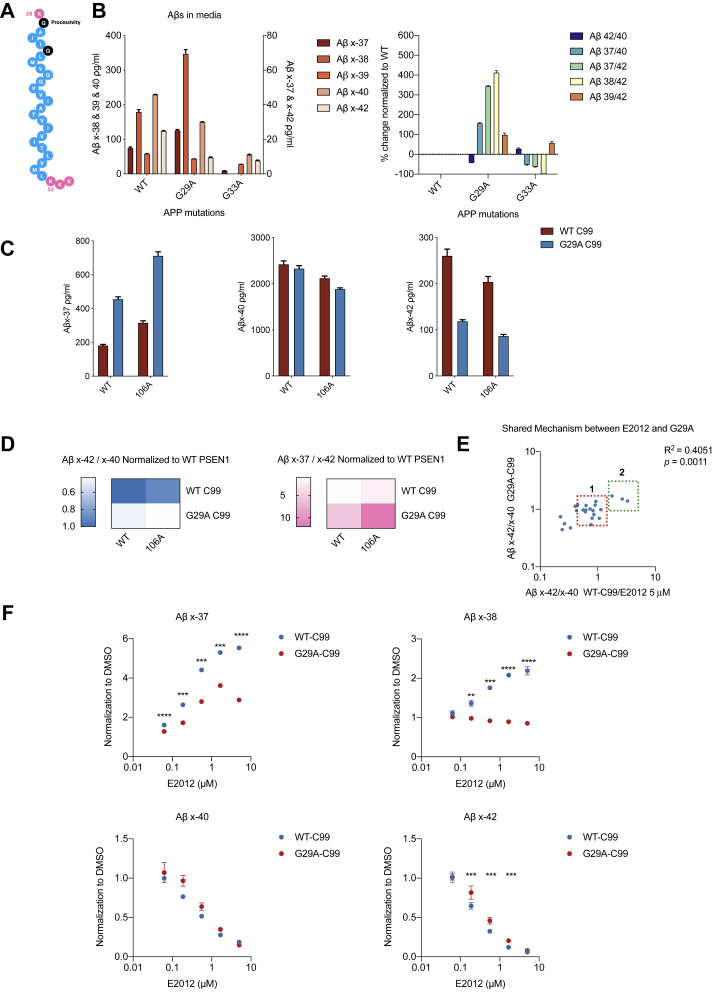
**Profile of Aβs from cells with G29A, G33A APP-C99.***A*, sequence of transmembrane domain of APP, where G29 and G33 are highlighted in black; (*B*) Aβ ELISAs on CM of HEK293 cells expressing WT, G29A or G33A C99. n = 3; (*C* and *D*) Aβ ELISAs on CM of dKO 293 cells coexpressing WT-PS1/WT-C99, WT-PS1/G29A-C99, Y106A-PS1/WT-C99, or Y106A-PS1/G29A-C99. n = 3. *E*, Aβ ELISAs on CM of dKO cells coexpressing WT-PS1 or each of 22 FAD PS1 mutations/WT-C99 treated with 5 μM E2012 (on the abscissa) or coexpressing WT-PS1 or 22 FAD PS1 mutations/G29A-C99 treated with DMSO (on the ordinate). Data normalized to WT-PS1/WT-C99-expressing cells with DMSO vehicle. Means of n = 3. *F*, Aβ ELISAs on CM of HEK293 cells expressing WT or G29A-C99 treated with serial doses of E2012. Data normalized to same cells treated with DMSO vehicle. Means of n = 3. Multiple paired *t*-test was used to compare the effect of each dose E2012 on WT or G29A-C99 expressing cells: ∗∗, *p* < 0.01; ∗∗∗, *p* < 0.001; ∗∗∗∗, *p* < 0.0001.

Allosteric modulation of the γ-secretase processing of APP into shorter Aβs involves the interactions of enzyme (presenilin) and substrate (APP). Multiple lines of evidence have also shown that γ-secretase processivity can be governed by the affinity between PS1 and APP. Within the juxtamembrane and transmembrane region of APP, there are three GxxxG motifs from amino acid 25 to 37 (Aβ numbering), a motif that was first reported to be important for possible dimerization of the TMD (20). Though recent cryo-EM (10) and biochemical (21) data have ruled out a dimerized APP TMD as an active substrate, the GxxxG motif has instead been reported to be important for γ-secretase processivity (21), in that the helix-breaking property of glycines could drive the premature release of longer Aβ peptides from the enzyme complex (relevant APP sequence is shown in Fig. 5*A*).

To focus on the processing of APP-βCTF into Aβ, we generated constructs to express this APP-C99 substrate (WT, G29A, and G33A). First, we expressed each of these three constructs in HEK293 cells and measured Aβs secreted into the CM (Fig. 5*B*, left panel). Compared with WT, G29A C99 led to dramatic increases in production of shorter Aβ peptides (Aβ37 and 38), decreased production of longer Aβs (Aβ40 and 42), and little change in Aβ39 production. This finding shows that G29A enhances γ-processivity, corroborating previous reports (20, 21). However, in contrast to earlier findings (20), G33A yielded much lower Aβ production overall. After calculating various ratios between Aβ peptides, we found G29A-C99 shares the same profile as Y106A-PS1, namely decreased Aβ42/40 ratio and increased Aβ37/40, 37/42, 38/42, and 39/42 ratios (Fig. 5*B*, right panel), indicating an improved processivity toward shorter peptides.

We further explored the complexity of enzyme/substrate interactions by expressing PS1 (WT or Y106A) with APP-C99 (WT or G29A) in the dKO cells (Fig. 5*C*). Here, we found that G29A-C99/Y106A-PS1 synergistically generated even more Aβ37 and less Aβ40 and 42 than either G29A-C99 or Y106A-PS1 alone. Thus, there was an unexpected amplification effect from altered substrate plus altered enzyme, each of which produces enhanced processivity alone, as represented by the further decrease in Aβ 42/40 ratio (42% of WT-C99/WT-PS1) and increase in Aβ 37/42 ratio (12-fold of WT-C99/WT-PS1), (Fig. 5*D*). This finding further indicates the importance of proper enzyme:substrate interaction for beneficial γ-secretase processing.

We then asked whether G29A-C99 and a GSM would act similarly on PS1. Here, we generated a library of 22 FAD PS1 mutations to test the effects of G29A-C99 or a GSM. Within the library, we included FAD mutants known to be resistant to GSMs (L166P and N135S) (22) and also the aforementioned T116R and L113P). The dKO cells were cotransfected with one of these 22 FAD PS1 mutants or WT PS1 together with a) WT-C99 (and treated with 5 μM E2012) or b) G29A-C99 (and treated with DMSO). The Aβ42/40 ratio from the WT-C99/WT-PS1/DMSO-treated cultures was normalized to 1, and the Aβ42/40 ratios from the two groups (a or b) were compared (Fig. 5*E*). We found that for the 22 mutations, G29A-C99 and E2012 showed similar Aβ effects in the presence of the PS1 FAD mutations (Pearson correlation *p*= 0.0011). We also identified 11 out of 22 mutations as having variable resistance to the effects of E2012, and 16 out of 22 mutations as having variable resistance to the effects of G29A-C99. When we set a criterion for this resistance of <25% lowering of the Aβ42/40 ratio by G29A-C99 or by E2012, we could categorize these resistant FAD mutations into two groups: group 1 with unchanged Aβ42/40 and group 2 with increased Aβ42/40 from either G29A-C99 or E2012 treatment (Fig. 5*E*). The surprising elevation of Aβ42/40 ratio produced by E2012 or by G29A-C99 resulted from reduced Aβ40 but no change in Aβ42 production (see Fig. S3). Moreover, through detailed profiling of the secretion of Aβ42 and 40 as well as 37, we found that G29A-C99 and E2012 treatment was actually effective for 21 out of 22 PS1 FAD mutations, in that G29A-C99 and E2012 only failed to increase Aβ37 production for L113P (Fig. S3). When using as the benchmark the Aβ37/42 ratio, E2012 was found to enhance processivity to varying degrees for all 22 FAD mutations (Fig. S4*A*, lower panel). A related question is whether enhanced processivity that still leaves Aβ42 unchanged could be therapeutically beneficial. In this regard, a recent *in vivo* study suggests that shorter Aβ’s, such as 37 and 38, could attenuate Aβ42 toxicity by reducing the aggregation propensity of the latter peptide (23). Therefore, enhanced production of Aβ37 will potentially be helpful even with a rare lack of an Aβ42-lowering effect for certain resistant FAD mutations.

We asked whether E2012 and G29A share the same mechanism of promoting γ-processivity. We compared the effects of using E2012 to treat cells expressing either WT or G29A-C99. As shown in Figure 5*F* and S4*B*, HEK293 cells transiently expressing WT or G29A-C99 were treated with serial doses of E2012 (from 67 nM to 5 μM). After normalization to the DMSO-treated control group, we found that E2012 could further decrease Aβ40 and 42, while increasing Aβ37 but not 38 in the G29A-C99 expressing cells (Fig. 5*F*). However, for the decrease of Aβ42 and increase of Aβ37, the G29A-C99 expressing cells are less sensitive to E2012 than were the WT-C99 expressing cells. The absolute values of the secreted Aβ peptides (Fig. S4*B*) show that the effect size on G29A-C99 is similar to a ∼550 nM dose of E2012 in reducing Aβ40 and 42, which is a remarkably strong effect (the E2012 EC_50_ was shown to be 146 nM in our system, Fig. 3). These data suggest that the effects of E2012 and G29A are shared mechanistically, in that G29A-C99 blunts the effects of E2012 on Aβ38.

Glycine residues in the APP ectodomain and TMD can be disease-promoting, as indirectly reflected by certain FAD APP mutations such as Aβ21G, Aβ22G, and Aβ45G (Fig. S5*A*). To extend this, we generated T43G-C99 and I45G-C99 and tested their effects on Aβ processing (Fig. S5, *B* and *C*). We observed two clear findings 1) 43G-C99 and 45G-C99 each strongly enhanced Aβ42 production; and 2) the processivity from Aβ42 to Aβ38 was not impaired and was even promoted. We discuss these interesting findings below.

In summary, our data here suggest that 1) G29 is a critical residue for γ-secretase processivity; 2) there is a synergistic effect between G29A-C99 and Y106A-PS1 in enhancing γ-secretase processivity; and 3) G29A can mimic the effects of the GSM E2012 on different PS1 FAD mutants.

## Discussion

γ-Processivity (carboxypeptidase-like trimming) is a critical function of γ-secretase, in which PS1 acts as the catalytic core. Substrate processing by γ-secretase is complicated, in that 1) it is not selective, as many substrates and different endo-proteolytic cleavage sites exist, *e.g.*, Aβ48 or Aβ49; 2) it involves multiple carboxyl-terminal proteolytic events, such as the Aβ48 or Aβ49 pathway toward Aβ38 or Aβ37, respectively; and 3) it involves premature product release of multiple Aβ intermediates. This last feature of γ-secretase is most critically dependent on processivity. Collectively, our data help illuminate the mechanism behind this complex process of product release.

As modeled by Okochi and colleagues (24), there are two key enzymatic rate kinetics, k_cat_ (catalytic rate constants) and k_b_ (dissociation rate constants), that govern each proteolytic step and the length of the Aβ peptides produced. k_cat_ determines the hydrolysis event, and k_b_ is a term that is dependent on the variable affinity between the substrate and the enzyme. To shift Aβ species toward shorter products under a constant k_cat_, the affinity between the enzyme (PS1) and substrate (C99 and the Aβ intermediates), reflected by k_b_, needs to be strengthened in this sequential proteolysis, in which the product from the last proteolytic event becomes the substrate for the next event, but only if the substrate is longer than 37 or 38 residues, since Aβ 37 and 38 can no longer associate with the enzyme and are released as final products. In other words, the relatively high k_b_ for Aβ_n_:γ-secretase (wild-type) is why we are able to detect secretion (release) of Aβ 37, 38, 39, 40, 42, and 43 into the brain extracellular fluid, including cerebrospinal fluid. But even for the wild-type γ-secretase, the multiple k_b_’s for the sequential enzymatic reactions are not sufficient to fully retain the substrates, since Aβ 37 and 38 (the shortest end products) are only present at around 20–30% of the total Aβ monomers released into human cerebrospinal fluid (25) (also supported by our unpublished data). Could the relative k_b_ for Aβ_n_:γ-secretase represent a vulnerability factor for AD? We investigated this question from the perspective of both the enzyme and the substrate.

For the enzyme, we first identified Y106A, an artificial variant, that can boost the processivity by all the benchmarks: Aβ42/40, Aβ37/40, Aβ37/42, and Aβ38/42. Several PS1 variants have been reported, including L383N (26), L241I, F411Y, S438P, F441L (27), V236C (28), and V236S (29), that could also enhance the γ-secretase processivity. Compared with these variants, Y106A demonstrated a more profound effect as it 1) simultaneously decreased long Aβ and increased short Aβ dramatically, and 2) generated similar amounts of total Aβ as WT PS1 did, indicating a pure boost in processivity instead of altering the initial endoproteolysis (ε-cleavages). So, we suspected that Y106 could be a key determining factor for k_cat_ and k_b_. For the substrate (C99), we examined G29A, an artificial variant, which again boosted processivity by all the benchmarks: Aβ42/40, Aβ37/40, Aβ37/42, and Aβ38/42. Consistent with the previous reports (20, 21), we show that G29 is a critical site on APP for γ-secretase processivity. Furthermore, our new data obtained with the novel Aβ immunoassays revealed further details on all of the secreted Aβ species. It has been shown that most of Notch-1 as a γ-secretase substrate can be processed into its primary final product (at the S4 cleavage site) that is analogous in substrate position to Aβ 36 (30). Also, we searched the predicted ectodomain and transmembrane sequences of 77 type-1 transmembrane substrates of γ-secretase (31). Among them, there are nine proteins containing a GxxxG domain (Jagged2, Syndecan-1, EphrinB2, LRP-2, LRP-6, EpCAM, and RPTP_*k*_ have 1 while RPTPμ has 2), and only APP has three repeated GxxxG domains. We speculate that this uniquely repeated motif in APP leads to an inevitable generation of long Aβs. If so, this concept could potentially answer the long-standing puzzle in the AD field: why the peptide products of other γ-secretase substrates do not aggregate like Aβ, even though those substrates contain transmembrane sequences with similar hydrophobicity as APP.

So, we wondered whether Aβ_n_ itself is a less than an optimal γ-secretase substrate because the repeated GxxxG domain (unique for APP) could represent a biochemical "defect" that leads to somewhat longer Aβ peptides, and due to the greater hydrophobicity of Aβ42 and 43 in particular, to Aβ deposition with age. It is likely that k_cat_ and k_b_ can independently affect Aβ processing, as we found that both 43G-C99 and 45G-C99 led to enhanced production of Aβ42 but even greater production of Aβ38. Thus, perhaps 43G and 45G solely increase k_b_ (substrate-based) without altering k_cat_. Analogous to how carriers of the Icelandic (A673T) APP mutation have a reduced risk for AD (32), we speculate that mutations in a GxxxG motif, such as our G29A, could serve as another way to lessen Aβ deposition and thus subsequent neurodegeneration and dementia. Perhaps this motif of APP could be targeted in APP transgenic mice, such as *via* gene therapy, to establish a proof of concept. In this regard, a previous report has shown successful lowering of Aβ production by a CRISPR/Cas9-based strategy that edits the C terminus of APP to shift APP processing from β- toward α-secretase processing (33). In comparison, targeting the G29 glycine residue could be a more precise and robust gene-editing therapeutic approach. The only change introduced would be that in γ-processivity toward APP, not toward other γ-substrates nor other enzymes, which process APP. In light of the development of new viral vectors for CNS delivery (34), we may proceed to explore the possibilities of gene editing of the GxxxG motif in mice as a targeted “Aβ-shortening” therapy.

We also further investigated the mechanisms of two classes of GSMs. First, we confirmed that the final products of γ-secretase are Aβ37 and 38, as all five GSMs tested, as well as the G29A substitution in C99, reduced Aβ 42, 40, and 39. Second, acidic GSMs and heterocyclic GSMs did not share the same Aβ profile, with JNJ-40418677 having intermediate effects between the two classes. Third, we present here evidence for Y106 as a binding site for the heterocyclic GSM, and we show that G29A-C99 (substrate) could further enhance the processivity of PS1 Y106A (enzyme), whereas E2012 and sGSM40 (heterocyclic GSMs) could not. We assumed that the Y106A PS1 lost its binding capacity to modulatory compounds but could still respond to a "better" (more processive) substrate: G29A-C99. A new structural study (35), published while the present study was under review, used cryo-EM to study the binding between γ-secretase and E2012 to reveal that the binding orientation of E2012 is stabilized by an H-bond between the methylimidazole and the side chain of Y106. This observed binding mode of E2012 is consistent with our biochemical data herein. Fourth, we found that the effects of E2012 and G29A-C99 on FAD PS1 are highly correlated (Fig. 5*E*), indicating that GSM and G29A may induce functionally similar conformational changes in the Aβ_n_:γ-secretase complex.

In summary, we present a detailed investigation of γ-secretase processivity on APP, using systematic mutagenesis of PS1 and APP-C99 together with five GSMs. We confirm the critical roles of both hydrophilic-loop 1 of PS1 and G29 in the APP TMD for γ-processivity in that Y106 is a binding site for heterocyclic GSMs; that Y106A-PS1 and G29A-C99 both enhance γ-processivity dramatically; and that heterocyclic GSMs such as E2012 may share a mechanism with G29A-C99 in enhancing γ-processivity. These findings should benefit the rational design of next-generation GSMs having higher specificity and potency to lower longer, more amyloidogenic Aβ peptides in human brain.

## Experimental procedure

### Antibodies and chemicals

The following antibodies were used: anti-α-Tubulin (T9026, Sigma), anti-PS1-N (E3L9X, CST), anti-PS1-C (EPY2000, Abcam), anti-PS2-C (EPY1515, Abcam), anti-APP (A8717, Sigma), and anti-NCT (D4F6N, CST). Among the GSMs, JNJ-40418677 was from Aobious, E2012 was from Tocris, GSM-1 was from MedKoo, and TC-E 5006 was from Tocris. sGSM40 was a generous gift from Dr Rudy Tanzi.

### Generation of Presenilin1/2 double knockout cell line (PS1/2 DKO)

Homozygous human Presenilin1/2 double-knockout HEK-293 cell lines were generated using a clustered regularly interspaced short palindromic repeat (CRISPR)/Cas9 nuclease-mediated system (Addgene). The guide RNAs were designed using the CRISPR Design software package (http://crispr.mit.edu/) to minimize potential off-target effects. Two oligo pairs for Presenilin1 and three oligo pairs for Presenilin 2 were cloned into vector PX459 (Addgene, #62988) to express five gRNAs targeting both Presenilin 1/2. After transfection of PX459 into HEK293 cells for 48 h, puromycin selection was undertaken for 1 week. Presenilin 1/2 double-knockout cells were isolated *via* limiting dilution cloning and confirmed by western blots.

### Generation of presenilin-1 and C99 expression vectors

PcDNA 3.1 vector harboring the wild-type human presenilin-1 and PcDNA 3.1 vector harboring the wild-type human APP-C99 with signal peptide 1–16 were used for the template to generate presenilin-1 and C99 expression vectors. To introduce mutations, the template was amplified by PCR into two DNA fragments with an overlapping sequence containing the mutated location. Overlap PCR was done to generate the whole open reading frame containing the mutation. PCR products were subcloned into the parental vector. Vectors were sequenced from both 5’ and 3’ ends to confirm successful mutagenesis.

### Tissue culture and transfection of adherent cells

Adherent HEK cells were cultured in complete growth media: Dulbecco’s Modified Eagle’s Medium (DMEM) supplemented with 10% fetal bovine serum (FBS), 2 mM L-glutamine, 10 units/ml penicillin, and 10 mg/ml streptomycin. For transfection, adherent HEK cells were seeded in 24-well dishes at a density of 5 x 10^5^ cells per well. Transfection was carried out with jetPrime reagent. Cells were incubated for 24 h and media were changed for conditioning after another 12 h, at which time the conditioned media were harvested for ELISA, and the cells were harvested for western blots.

### Aβ ELISA

Conditioned media from transfected HEK cells were harvested and diluted with 1% BSA in wash buffer (TBS supplemented with 0.05% Tween). For Aβx-37, x-38, x-39, x-40, x-42, and x-43 assays, each well of an uncoated 96-well multiarray plate (Meso Scale Discovery, #L15XA-3) was coated with 30 uL of a PBS solution containing 3 μg/ml of 266 capture antibody (Elan) and incubated at room temperature overnight. A detection antibody solution was prepared with biotinylated monoclonal antibody recognizing the respective C-terminal residue of each Aβ peptide, plus 100 ng/ml Streptavidin Sulfo-TAG (Meso Scale Discovery, #R32AD-5) and 1% BSA diluted in wash buffer. Following overnight incubation, 50 μl/well of the CM sample and 25 μl/well of the detection antibody solution were incubated for 2 h at room temperature with shaking at >300 rpm, washing wells with wash buffer between incubations. The plate was read and analyzed according to manufacturer’s protocol.

## Data availability

All data are contained within the article.

## Supporting information

This article contains supporting information.

## Conflict of interest

The authors declare that they have no conflicts of interest with the contents of this article.
