# Supplementary material for: Hydrophilic loop 1 of Presenilin-1 and the APP GxxxG transmembrane motif regulate γ-secretase function in generating Alzheimer-causing Aβ peptides
Source: J Biol Chem. 2021 Feb 8;296:100393. doi: 10.1016/j.jbc.2021.100393 (PMC7961089; doi:10.1016/j.jbc.2021.100393)
Supplement: Figures S1–S5 [file mmc1.pdf]

Supplemental Information

Hydrophilic loop 1 of Presenilin-1 and the APP GxxxG transmembrane motif regulate γ-secretase function in generating Alzheimer-causing Aβ peptides

Lei Liu, Bianca M. Lauro, Michael S. Wolfe and Dennis J. Selkoe

Supplemental figure 1

Supplemental figure 2

Supplemental figure 3

Supplemental figure 4

Supplemental figure 5

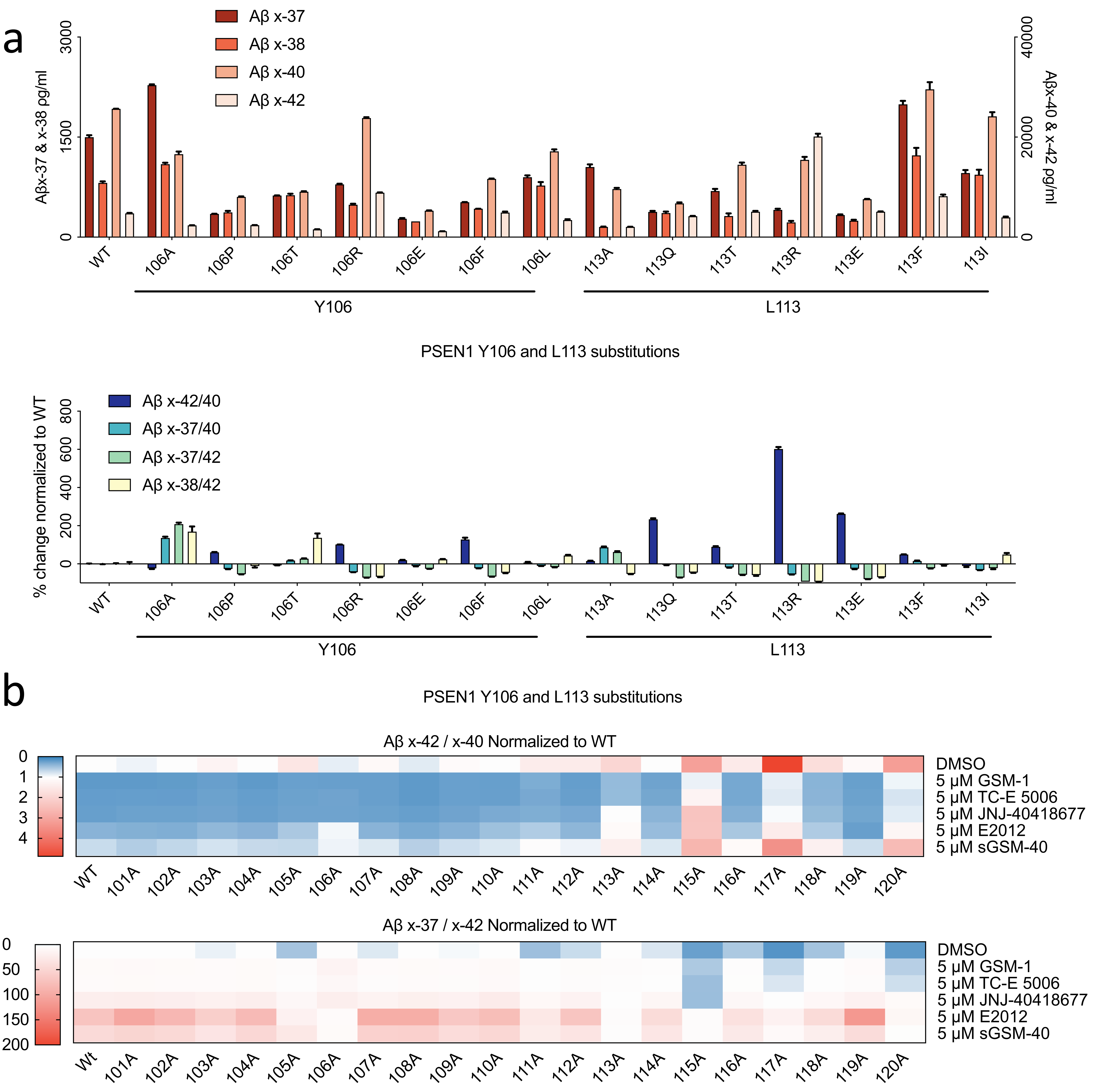

Figure S1: (a) Aβs measured by ELISA from CM of dKO cells co-expressing wt or PS1 variants plus WT APP (means ± SD, n=3). Upper panel: left Y-axis: Aβx-37 and 38, right Y-axis: Aβx-40 and 42. Lower panel: Aβ peptide ratios.
(b) Aβx-42/40 & 37/42 ratios from Aβ levels in (Fig.4) and normalized to control (wt PS1/DMSO) (white) with lower value in blue & higher value in red.

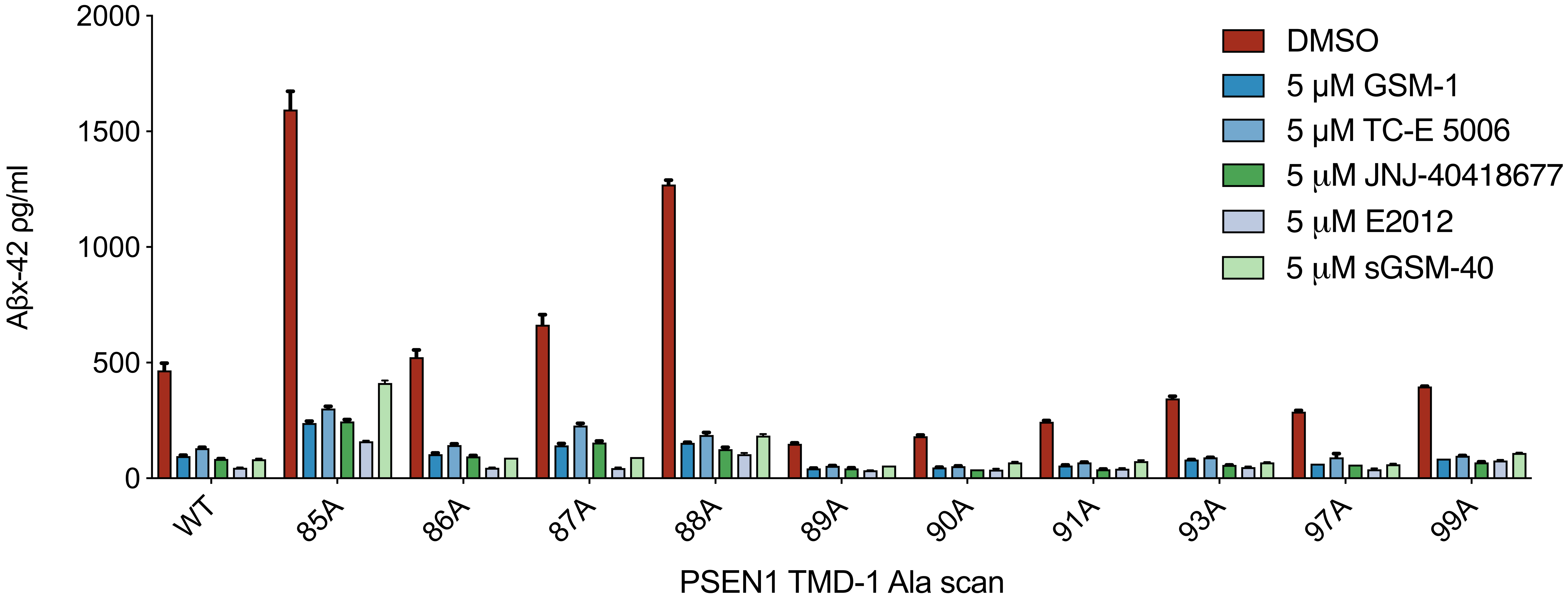

Figure S2: Profile of secreted Aβ42 from cells expressing Ala-substituted PS1 variants of TMD-1 region (n=3).

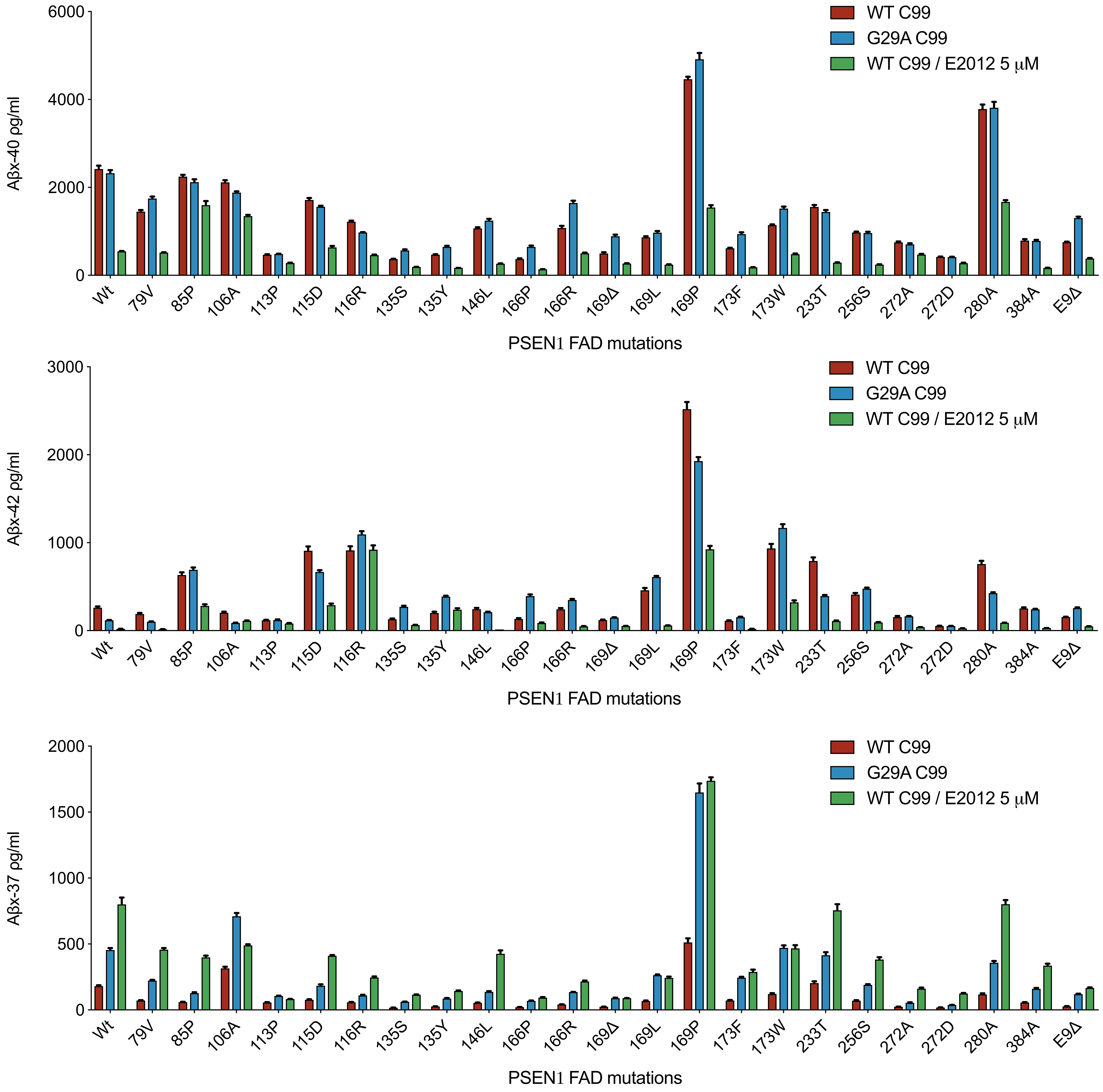

Figure S3: Profile of secreted Aβs from cells expressing FAD PS1 mutations with G29A-C99 or E2012 (n=3).

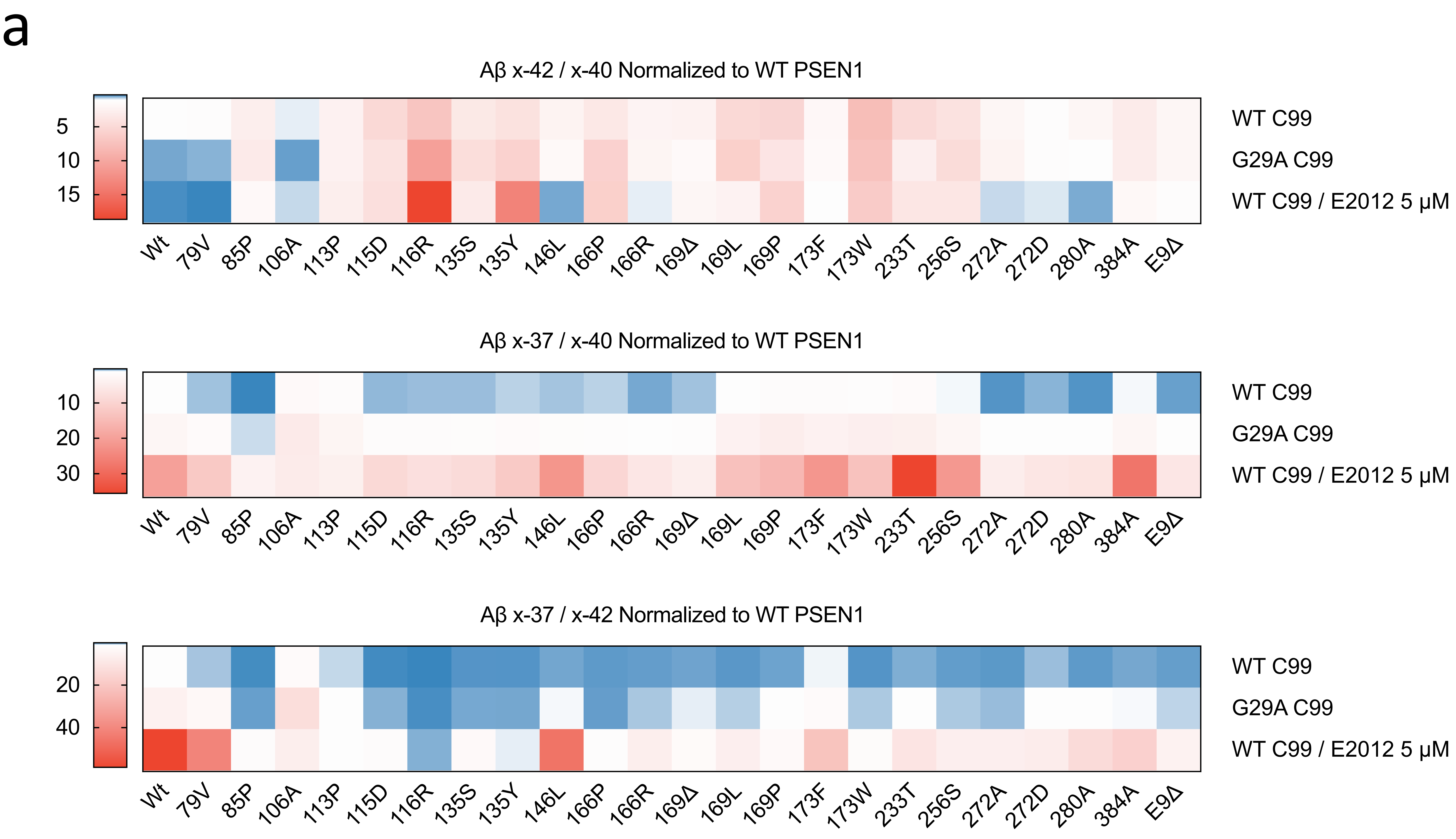

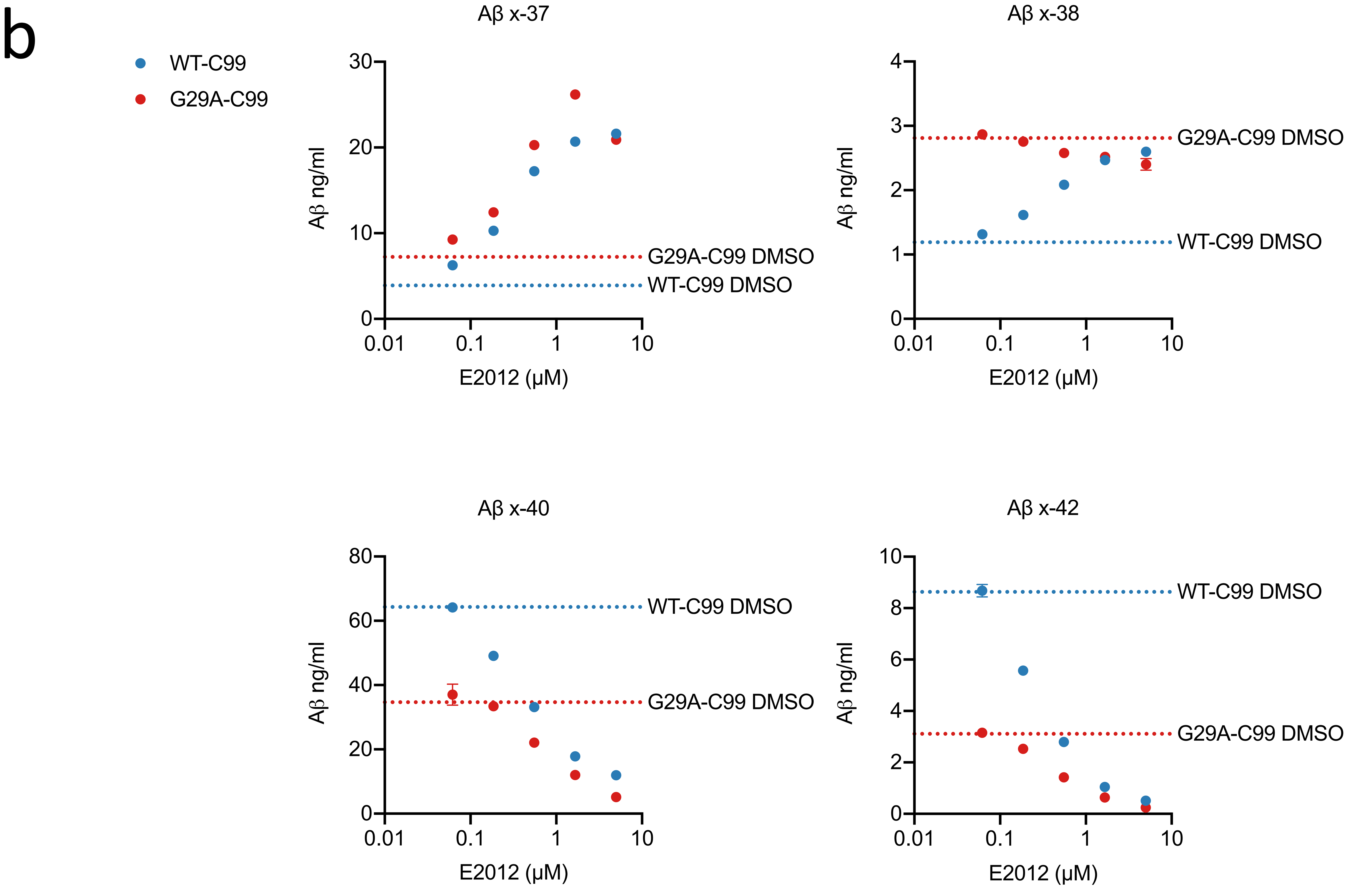

Figure S4: (a) Aβx-42/40, 37/40 and 37/42 ratios from Aβ levels in (Supplementary Fig.3) and normalized to control (wt PS1/DMSO) (white) with lower value in blue & higher value in red. (b) Absolute values of Aβ ELISAs on CM of HEK293 cells expressing WT or G29A-C99 treated with a serial dose of E2012 in (Fig. 5f). Means of n=3.

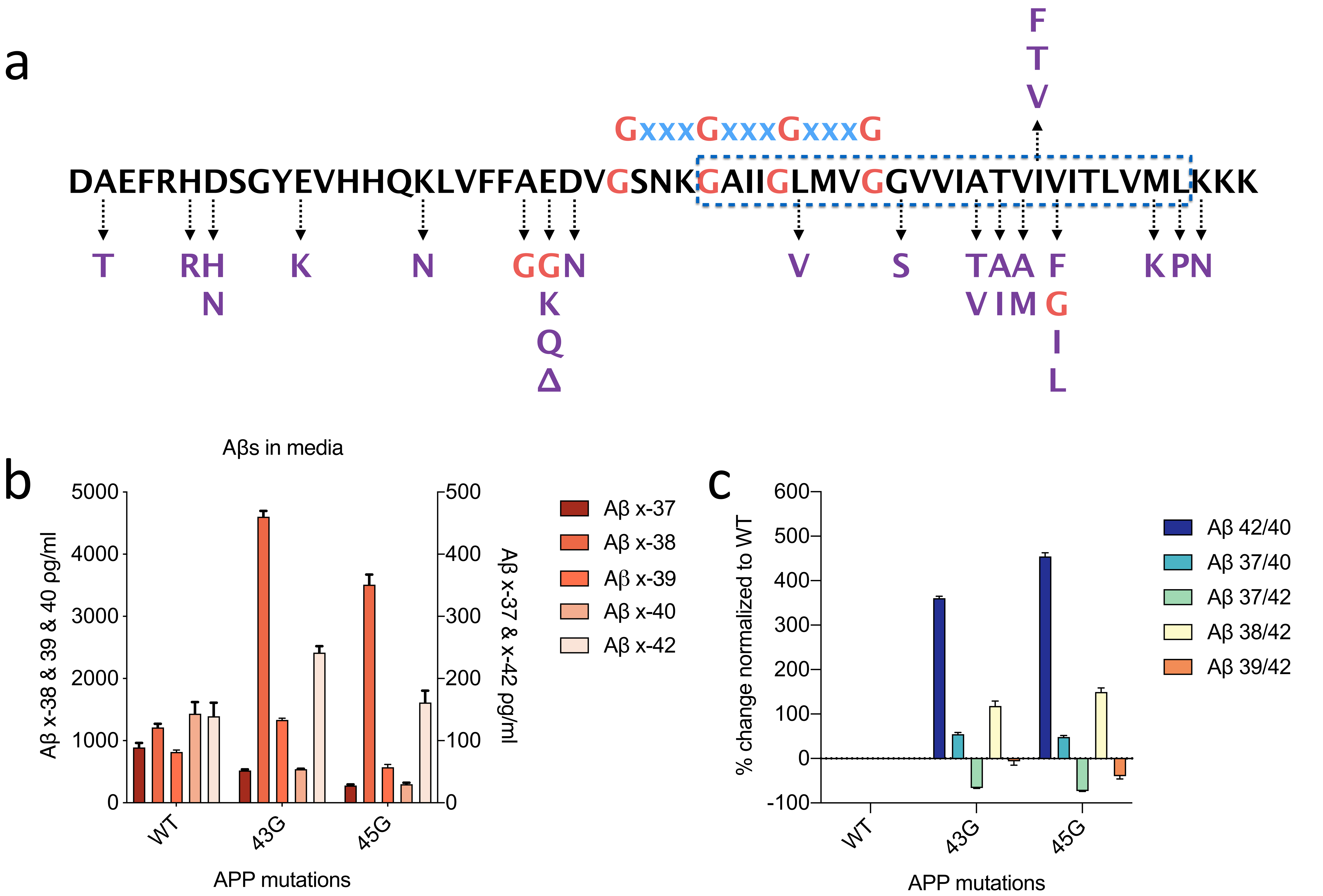

Figure S5: Profile of secreted Aβs from cells expressing T43G and I45G-C99. (a) Protein sequence of ectodomain and transmembrane domain of APP, while the both the GxxxG repeat motifs and FAD mutations were listed, Glycine residue is highlighted in red. (b-c) Profile of secreted Aβs from cells expressing 43G and 45G-C99, n=3, mean +/- SD.
